# Characterization of the complete mitochondrial genome of Zhenhai brown frog *Rana zhenhaiensis* (Anura: Ranidae)

**DOI:** 10.1080/23802359.2019.1667897

**Published:** 2019-09-23

**Authors:** Min-Yi Huang, Qiang Zhao, Yan-Qing Wu

**Affiliations:** a College of Agriculture and Biotechnology, Hunan University of Humanities, Science and Technology, Loudi, PR China;; b Ministry of Ecology and Environment, Nanjing Institute of Environmental Sciences, Nanjing, Jiangsu, PR China

**Keywords:** *Rana zhenhaiensis*, mitochondrial genome, phylogenetic analysis

## Abstract

*Rana zhenhaiensis* is a species of frog within the family Ranidae. In this study, its complete mitochondrial genome was characterized by high-throughput sequencing technology. It is 19,205 bp long with an overall AT content of 55.2% and includes 13 protein-coding genes (PCGs), 22 tRNA genes, 2 rRNA genes, and 1 control region. Besides, a 26-bp-long origin of L-strand replication (OL) is present between *tRNA-Asn and tRNA-Cys.* Our findings will be useful for the detailed study of mitogenome evolution and the phylogenetic relationships of the genus *Rana* and related taxa.

The Zhenhai brown frog (*Rana zhenhaiensis*) is a species of frog within the family Ranidae. It is widely distributed in central and southern China from sea level to 1800 m asl and plays an important role in maintaining the balance of farmland ecosystem (Xu et al. 2012; Wei et al. 2015). The complete mitochondrial genome of *R. zhenhaiensis* was characterized by high-throughput Illumina sequencing technology.

A sample of *R. zhenhaiensis* was collected from Dongzhi County, Chizhou City, Anhui Province in China (117°2′9″E, 30°18′6″N) with voucher specimen deposited at the biological museum of Hunan University of Humanities, Science and Technology (RW 2018061202). The genomic DNA extraction, library preparation, and Illumina sequencing were done by nanorod gene technology (Beijing, China). The high-throughput sequence data were then used for mitogenome assembly with the program MITObim v1.9 (Hahn et al. 2013). The mitogenome of *Rana kunyuensis* (KF840516) (Li et al. 2016) was used as the initial reference.

The mitochondrial genome of *R. zhenhaiensis* (GenBank accession MN 218687) is 19,205 bp in length and contains 13 protein-coding genes (PCGs), two rRNA genes, 22 tRNA genes, and 1 control region. The nucleotide composition is 27.5%A, 27.7% T, 14.9% G and 29.9% C, with an overall GC content of 44.8%. The length of origin of L-strand replication (OL) is 26 bp long, and is located between *tRNA^Asn^* and *tRNA^Cys^* (19,048–19,073). The *ND6* gene and eight tRNA genes (*tRNA^Ser^*, *tRNA^Glu^*, *tRNA^Pro^*, *tRNA^Gln^*, *tRNA^Ala^*, *tRNA^Asn^*, *tRNA^Cys^,* and *tRNA^Tyr^*) are encoded on the L-strand and the other genes on the H-strand. The start codons of PCGs are mostly ATG, except that *COX1* and *ND4L* begin with GTG. However, the start codon of *ND1* is yet to be determined, as similarly reported in *Rana chaochiaoensis* and *Rana omeimontis* (Yang et al. 2018). In all, five types of stop codon were annotated, i.e. AGG for *COX1* and *ND6*, TAG for *CYTB*, AGA for *ND5*, TAA for *ATP8* and *ND4L*, and an incomplete stop codon T for the remaining seven PCGs (*COX2*, *ATP6*, *COX3*, *ND3*, *ND4*, *ND1,* and *ND2*), which is presumably completed as TAA by post-transcriptional polyadenylation (Ojala et al. 1981). The 22 tRNA genes have a size range from 65 bp in *tRNA^Cys^* to 73 bp in *tRNA^Asn^* and *tRNA^Leu^*. The 12S and 16S rRNA genes located between *tRNA^Phe^* (70 bp) and *tRNA^Val^* (69 bp) are 930 bp and 1576 bp long, respectively.

To further analyze its phylogenetic position within the genus *Rana*, a maximum-likelihood (ML) phylogeny was built based on the 13 PCGs for a group of 19 *Rana* species with the program MEGA7 (Kumar et al. 2016) (Figure 1). Another two Ranidae species (*Amolops mantzorum* and *Amolops wuyiensis*) were included as the outgroup taxa. The phylogenetic analysis suggested that *R. zhenhaiensis* is closely related to *Rana omeimontis*.

**Figure 1.**
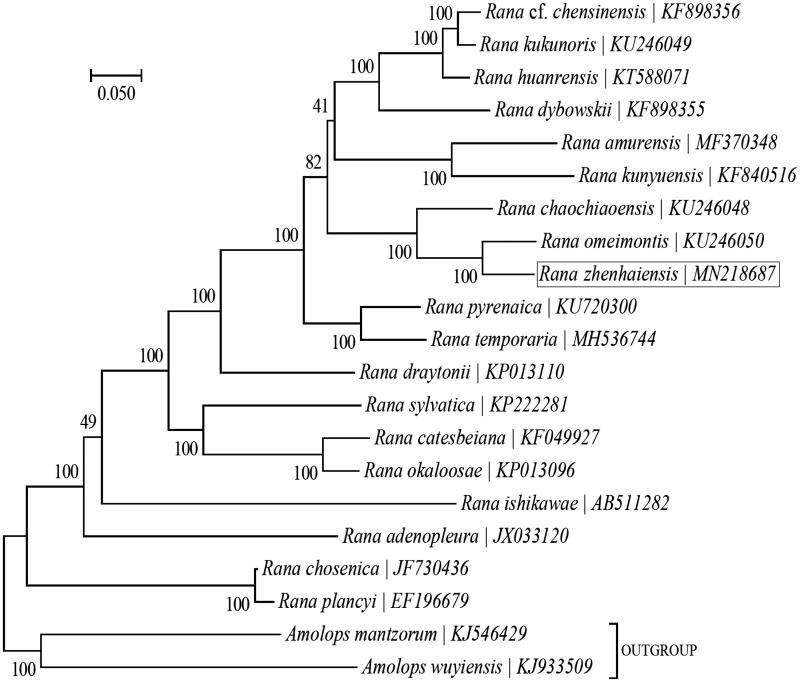
Phylogeny of the genus Rana based on the maximum-likelihood analysis of 13 mitochondrial PCGs. The best-fit substitution model is ‘GTR + G+I’. The bootstrap values are based on 100 random replicates. Genbank accession number for each species is shown after the species name.
